# Mode hybridization analysis in thin film lithium niobate strip multimode waveguides

**DOI:** 10.1038/s41598-020-73936-x

**Published:** 2020-10-07

**Authors:** Archana Kaushalram, Gopalkrishna Hegde, Srinivas Talabattula

**Affiliations:** 1 Electrical Communication Engineering, Indian Institute of Science, Bangalore, 560012 India; 2 Center for BioSystems Science and Engineering, Indian Institute of Science, Bangalore, 560012 India

**Keywords:** Integrated optics, Nanophotonics and plasmonics

## Abstract

Mode hybridization phenomenon in air-cladded X-cut Y-propagating and Z-propagating thin film lithium niobate strip multimode waveguides is numerically studied and a mathematical relation between structural parameters leading to hybrid modes is formulated. Dependence of hybrid modes on waveguide dimensions, sidewall angles and wavelength is also analyzed. The results obtained are used to design lithium niobate on insulator (LNOI) taper for converting fundamental TM mode to higher order TE mode, and an optimum length for achieving a high conversion efficiency of 99.5% is evaluated. Birefringent Y-propagating LN and isotropic Z-propagating LN tapers are compared in terms of length, figures of merit, and fabrication tolerance. Tapers exhibit a broad bandwidth of 200 nm with an extinction ratio less than − 18 dB. The results of mode hybridization analysis are useful in design optimization of adiabatic tapers, tunable time delays, optical interconnects, mode converters and demultiplexers for mode division multiplexing (MDM) applications.

## Introduction

Photonic devices on high-contrast platforms have guided modes that are inherently hybrid in nature^1^. They have all six field components (Ex, Ey, Ez, Hx, Hy, Hz) to be non-zero, unlike the planar waveguides which have pure transverse electric (TE) and transverse magnetic (TM) modes. However, there is a dominant E and a dominant H component for all the modes in general. Such modes are referred to as quasi-transverse electric (qTE) and quasi-transverse magnetic (qTM) modes. If high-contrast waveguides like silicon-on-insulator (SOI) and Lithium niobate on insulator (LNOI) have vertical asymmetry of index and/or horizontal asymmetry in structure, then there are some special modes where the dominant and non-dominant field components are almost equal^2,3^. Such modes cannot be classified as qTE or qTM modes and are called as “Hybrid modes”. This phenomenon of mode hybridization happens when two orthogonally polarized modes with similar effective indices couple to each other^4^. Mode hybridization regions are used in designing tapers for TM–TE higher order mode conversions^5–9^ in polarization splitter–rotators. These components are essential for the implementation of polarization diversity schemes, which circumvent the problem of polarization-dependent losses and dispersion in high-contrast waveguides^10^. They are also key components in optoelectronic integrated circuits for coherent optical system applications^11^. Hybrid modes are also reported to exhibit large group velocity dispersion compared to qTE and qTM modes, which could be useful in tunable time delays and optical signal processing applications^12^. While hybridization phenomenon is useful for mode converters and delays, the same could cause high-crosstalk in mode division multiplexing (MDM) applications^13–15^. Hybrid modes in SOI nanowires and LNOI are observed and reported in^4,9^. However, mathematical relation between structural parameters of waveguides that lead to hybrid modes and, its dependence on operating wavelength have not been explored yet.

In this paper, a complete analysis of mode hybridizations has been carried out in thin film lithium niobate (LN) strip multimode waveguides on X-cut Y-propagating and Z-propagating crystals. LNOI is an excellent platform for integrated photonics because of its broad transparency range from 350 to 5200 nm, strong electro-optic, acousto-optic, and thermo-optic effects which make it an excellent choice for active and non-linear devices as well^16–19^. Further, an attempt has been made to formulate a mathematical relation between structural parameters of waveguides that lead to hybrid modes. Dependence of this phenomenon on the operating wavelength and sidewall angles of waveguides are also studied. Based on the results obtained, tapers are designed on both birefringent and isotropic LN waveguides to convert TM_0_ to TE_1_ mode and, an optimum length is determined to achieve a large conversion efficiency of 99.5%. Device length and fabrication tolerance of mode converters that strongly depend on shift in hybrid points resulting from fabrication errors like sidewall angles and width deviations are also studied in detail.

**Figure 1:**
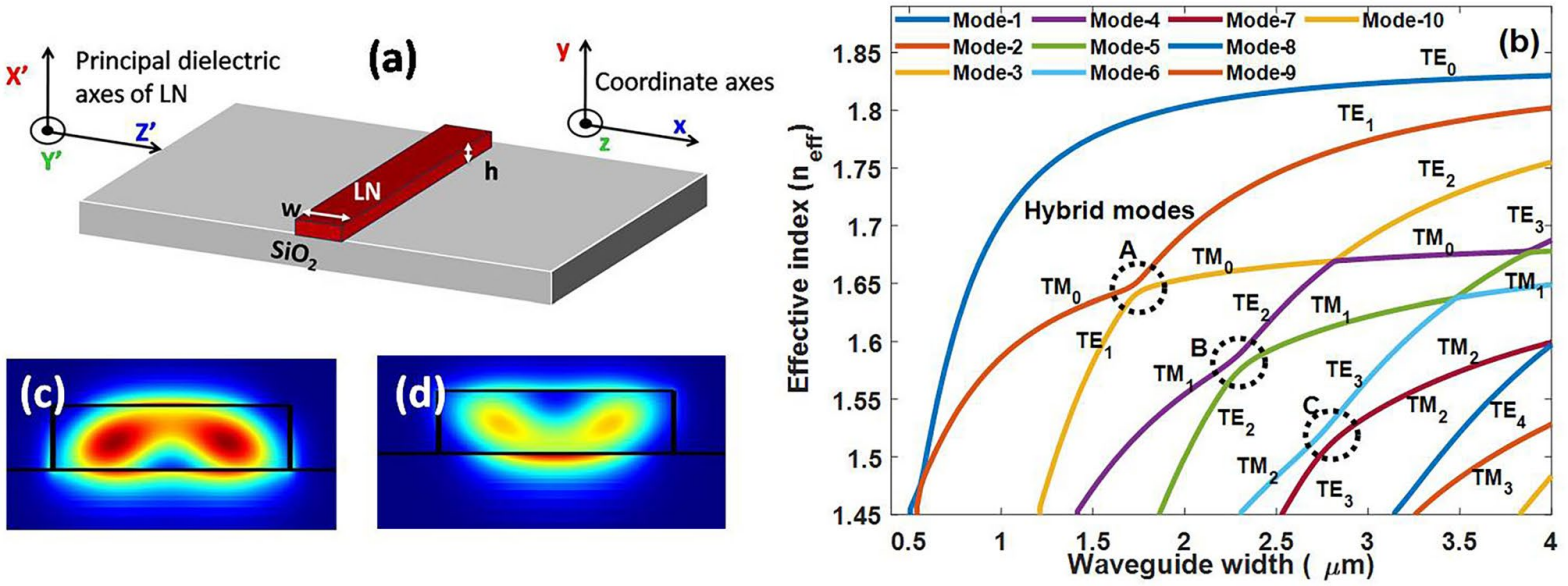
(**a**) Cross section of LNOI strip waveguide. Principal dielectric axes of X-cut Y-propagating LN and simulation axes. (**b**) Variation of effective index of modes with waveguide width in X-cut LNOI strip waveguide, black circles indicate hybrid modes. (**c**, **d**) Field profile of hybrid modes between TM_0_ and TE_1_ at hybrid point A. [1(b), (c), and (d)-Lumerical 2020a MODE Finite Difference IDE, version: 7.15.2152, release: 2020a r2, URL: www.lumerical.com] [Image converted to JPG file with Adobe Photoshop, Version: 21.2.1 20200716.r.265, URL: www.adobe.com].

The cross-section of the waveguide analyzed is shown in Fig. 1a, along with the chosen orientation for LN principal dielectric axes with respect to the waveguide. The dispersive nature of LN is modeled using Sellmeier equation^20^. Multimode waveguides are designed at a telecommunication wavelength λ of 1550 nm to support eight modes that include qTE, qTM, and hybrid modes at some special dimensions. The SiO_2_ buried oxide (BOX) layer is generally thick enough (2–3 µm) to avoid any field penetration into the underlying substrate (not shown here). Sample results for variation of effective index with waveguide width at a height of 0.4 µm is shown in Fig. 1b. The anticrossings marked in black dotted circles, where the effective indices of two orthogonally polarized modes become almost equal indicate hybrid modes. Field profiles of hybrid modes between TM_0_ and TE_1_ at hybrid point A are shown in Fig. 1c,d.

**Figure 2:**
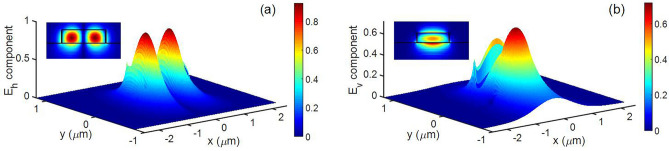
Field profiles of a hybrid mode between TM_0_ and TE_1_ in LNOI strip waveguide (a) Horizontal component (**b**) Vertical component. Both the components are almost equal indicating the mode is hybrid. [Lumerical 2020a MODE Finite Difference IDE, version: 7.15.2152, release: 2020a r2, URL: www.lumerical.com] [Image converted to JPG file with Adobe Photoshop, Version: 21.2.1 20200716.r.265, URL: www.adobe.com].

The horizontal (E_h_) and vertical (E_v_) electric field components for hybrid mode at point A are shown in Fig. 2 and is observed that E_h_ and E_v_ are almost equal. The phenomenon of hybridization can occur due to perturbation in the form of asymmetry in waveguide geometry^11^, or dielectric perturbation with the upper and the lower clad having different refractive indices^3^. The two hybrid super modes couple with each other at the anticrossing points, with a coupling factor (k) given by Eq. (1),

1 $$k = \frac{{\beta_{S1} - \beta_{S2} }}{2} = \frac{\Delta \beta }{2}$$

where β_S1_ and β_S2_ are the propagation constants of hybrid modes at the anticrossing point.

In X-cut Y-propagating LN, qTE modes see an extraordinary index (n_e_), while qTM modes see an ordinary index (n_o_), which results in birefringence or velocity difference between these modes. In X-cut Z-propagating LN, with Z being the optic axis of the crystal, both qTE and qTM modes see an ordinary index (n_o_), which is similar to isotropic material. Modal hybridness is quantized by a parameter called TE polarization fraction (γ_TE_), which indicates the fraction of power in the horizontal component of a mode^6^, as in Eq. (2),

2 $$\gamma_{TE} { } = { }\frac{{\smallint \left| {{\text{E}}_{{\text{x}}} } \right|^{2} dxdy}}{{\smallint \left( {\left| {E_{x} } \right|^{2} + \left| {E_{y} } \right|^{2} } \right)dxdy}}$$

when $\gamma_{TE}$ is close to 1, it represents a qTE mode, while a value close to 0 represents a qTM mode. TE polarization fraction of 0.5 represents strong hybridness of modes. In this analysis, a mode is considered hybrid if 0.4 < γ_TE_ < 0.6.

It is interesting to obtain physical intuitive insights into the nature of the hybrid modes as explained next***. ***In order to excite hybrid modes, the non-dominant (minor) field component needs to be enhanced. Existing approaches to increase the non-dominant field component include the following: setting the waveguide width equal to waveguide height^1^, introducing bends in the waveguide^21^, using index asymmetry in the vertical direction^3^, and using structural asymmetry like one sided angled side wall^11^. In this work, index asymmetry is present in the vertical direction as the strip waveguide has the top air clad and the SiO_2_ bottom clad. This can be treated as a buried waveguide (same top and bottom clad) with dielectric perturbation Δε.

Mode coupling between two modes occurs when the modes have similar effective indices, significant overlap between the mode profiles, and same symmetry for the individual field components. These conditions lead to a coupling factor^22^ defined by Eq. (3),

3 $$k = k_{x} + k_{y} + k_{z}$$

4 $$k = \frac{{\omega \varepsilon_{0} }}{4}{\iint }\Delta \varepsilon_{r} \underbrace{{E_{xi} E_{xj}^{*} }}_{Vx}dx dy + \frac{{\omega \varepsilon_{0} }}{4}{\iint }\Delta \varepsilon_{r} \underbrace {{E_{xi} E_{xj}^{*} }}_{Vy}dx dy + \frac{{\omega \varepsilon_{0} }}{4}{\iint }\frac{{\varepsilon_{r} \Delta \varepsilon_{r} }}{{\varepsilon_{r} + \Delta \varepsilon_{r} }}\underbrace {{E_{zi} E_{zj}^{*} }}_{Vz}dx dy$$

where indices i and j represent the modes being coupled.

**Figure 3:**
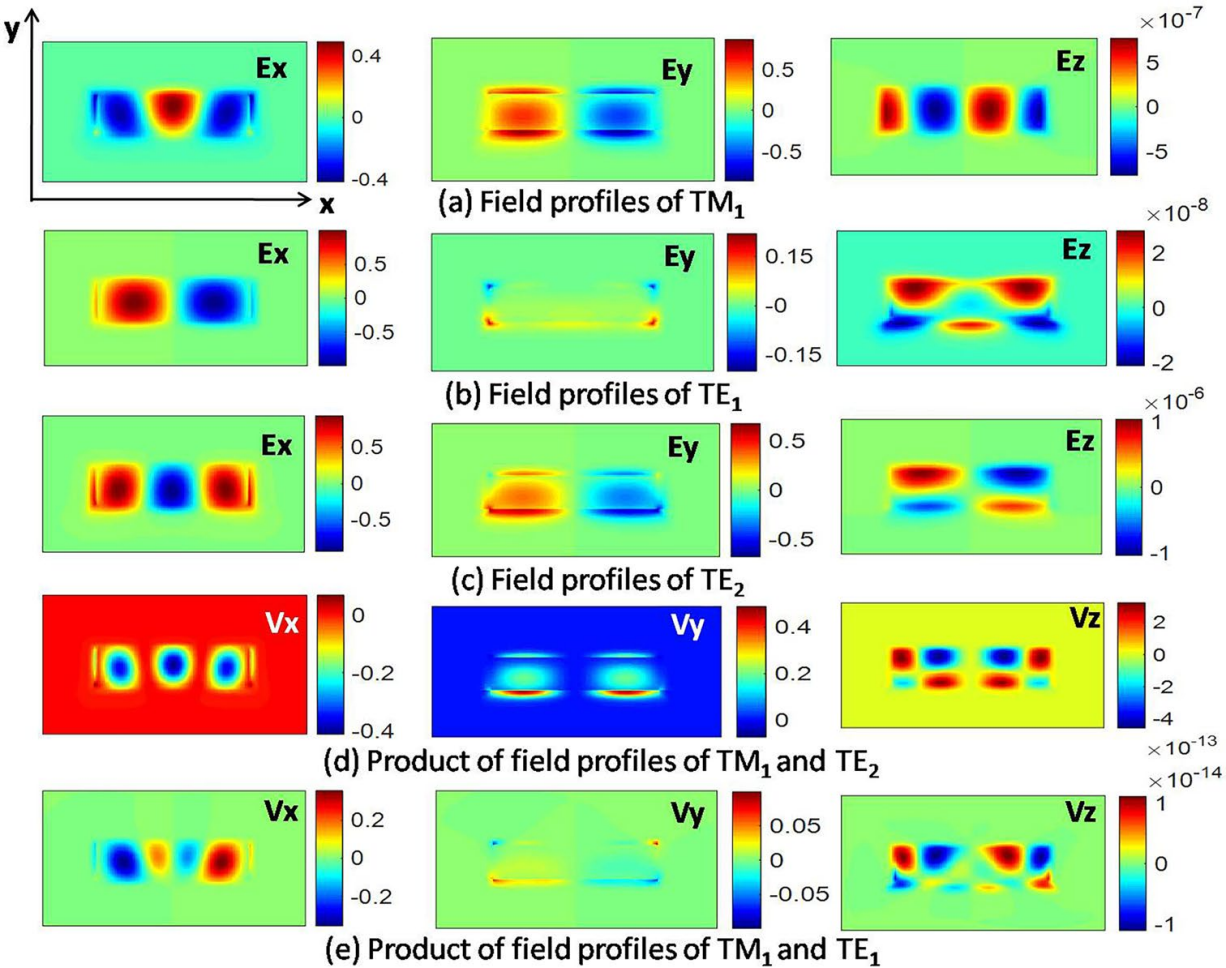
Field products V between different modes. Products with odd symmetric profile about y-axis will make the coupling factor zero. [Lumerical 2020a MODE Finite Difference IDE, version: 7.15.2152, release: 2020a r2, URL: www.lumerical.com] [Image converted to JPG file with Adobe Photoshop, Version: 21.2.1 20200716.r.265, URL: www.adobe.com].

Consider the example of a mode pair TM_1_ and TE_2_, with the waveguide width 2.2 µm and height of 0.35 µm on X-cut Y-propagating LN. These two modes become fully hybrid supermodes when the width is 2.135 µm. The individual field components of TM_1,_ TE_1_, and TE_2_ modes in this waveguide are shown in Fig. 3a–c. It can be observed that the respective field components of TM_1_ and TE_2_ have same symmetry. Hence for these two modes, the scalar products in Eq. (4) have an even symmetry with respect to y-axis (Fig. 3d), resulting in a non-zero coupling factor k. Next pair of modes TM_1_ and TE_1_ can also be observed for the possibility of mode coupling. It is seen that coupling factor k vanishes for this pair, as the scalar products in Eq. (4) exhibit odd symmetry with respect to y-axis about the waveguide center (Fig. 3e). While perturbation theory can be used to understand the physics of hybrid modes, supermode theory should be used to find accurate coupling factors for larger values of Δε.

With perturbation in the form of index asymmetry along vertical direction, this structure can lead to hybrids between TM_i_ and TE_i+2 k+1_, where i and k are integers. A structure with perturbation in the horizontal direction^23^ as in a L-waveguide can lead to hybrids between TM_i_ and TE_i+2 k_ as well. Another important criterion for mode coupling is the overlap factor^24^ which represents the fraction of electromagnetic fields overlapping between the field profiles of two modes as defined in Eq. (5). For the pair of modes where the coupling was possible (TM_i_ and TE_i+2 k+1_) in this waveguide structure, overlap factor was of the order of 10^−5^, while it was 10^−30^ for the hybridization-incompatible mode pairs.

5 $$overlap = \left| {Re\left[ {\frac{{\left( {\smallint {\varvec{E}}_{1} \times {\varvec{H}}_{2}^{*} .d{\varvec{s}}} \right)\left( {\smallint {\varvec{E}}_{2} \times {\varvec{H}}_{1}^{*} .d{\varvec{s}}} \right)}}{{\smallint {\varvec{E}}_{1} \times {\varvec{H}}_{1}^{*} .d{\varvec{s}}}}} \right]\frac{1}{{Re\left( {\smallint {\varvec{E}}_{2} \times {\varvec{H}}_{2}^{*} .d{\varvec{s}}} \right)}}} \right|$$

## Results and discussion: mode hybridization in LN strip waveguides

**Figure 4:**
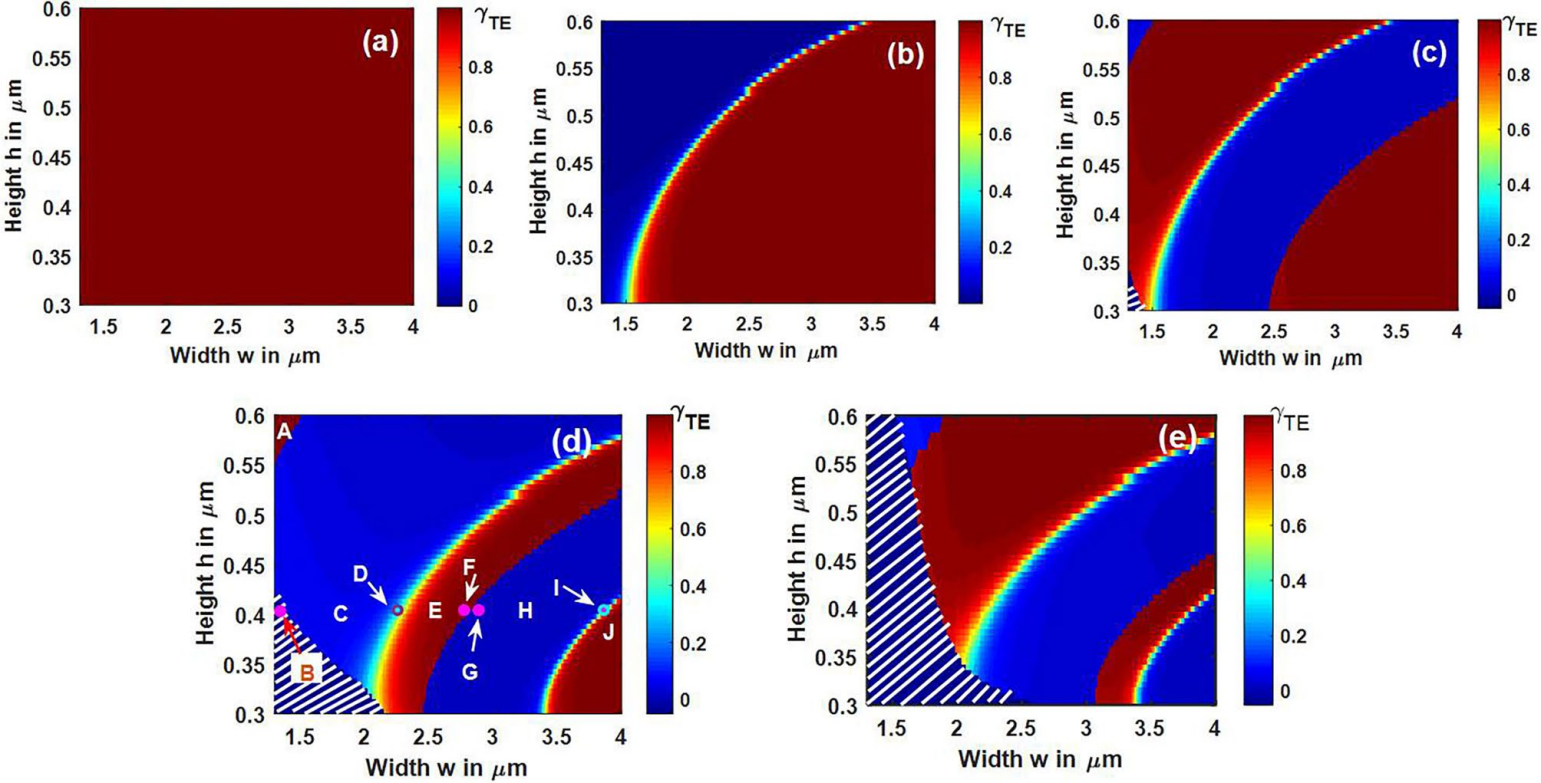
TE polarization fraction of (**a**) Mode-1 (**b**) Mode-2 (**c**) Mode-3 (**d**) Mode-4 (**e**) Mode-5 as a function of dimensions of X-cut Y-propagating LNOI strip waveguide. Green represents maximum hybridness. [Lumerical 2020a MODE Finite Difference IDE, version: 7.15.2152, release: 2020a r2, URL: www.lumerical.com] [Image converted to JPG file with Adobe Photoshop, Version: 21.2.1 20200716.r.265, URL: www.adobe.com].

The width ‘w’ of the strip waveguide is varied up to 4 µm and height ‘h’ between 0.3 and 0.6 µm to observe hybrid modes. Maximum height is restricted to 0.6 µm so as to have one peak in the vertical direction, with higher order modes supported along the horizontal direction. The dependence of TE polarization fraction on width and height of the X-cut Y-propagating LNOI waveguide for the first five modes at a wavelength of 1550 nm are shown in Fig. 4. The color bar shows that a qTE mode is represented by 1 (maroon at the top), qTM mode by 0 (navy blue at the bottom), and hybrid modes by cyan to yellow corresponding to 0.4 < γ_TE_ < 0.6. Green on the color bar indicates strongest hybridness. Mode-1 is the fundamental TE mode and remains so over the entire range of width and height variation. This is indicated by a maroon block in Fig. 4a. Modes 2 and 3 represent TM_0_ and TE_1_ just before the beginning of hybridization. White-striped regions in Fig. 4c–e represent cutoff regions for the corresponding modes. TE polarization fraction plots for modes 3, 4, and 5 in Fig. 4c–e show alternate maroon and blue strips apart from the hybridization curve (in green–yellow), because of change in the order of modes as the waveguide dimensions change. This is explained for mode-4 in Table 1, where different sample pairs of (w, h) are considered in each region.

**Table 1:** Sample points from Fig. 4(d) of mode-4 indicating transition from qTE to qTM and then to hybrid mode.

Sample points (w,h) in μm	Mode-1	Mode-2	Mode-3	Mode-4	Mode-5
A(1.376, 0.5898)	TE_0_	TM_0_	TM_1_	TE_1_	Cutoff
B(1.365, 0.4017)	TE_0_	TM_0_	TE_1_	Cutoff	Cutoff
C(1.896, 0.4017)	TE_0_	TE_1_	TM_0_	TM_1_	TE_2_
D(2.308, 0.4017)	TE_0_	TE_1_	TM_0_	Hybrid	Hybrid
E(2. 500, 0.4017)	TE_0_	TE_1_	TM_0_	TE_2_	TM_1_
F(2.829, 0.4017)	TE_0_	TE_1_	TE_2_	TM_0_	TM_1_
G(2.840, 0.4017)	TE_0_	TE_1_	TE_2_	TM_0_	TM_1_
H(3.317, 0.4017)	TE_0_	TE_1_	TE_2_	TM_0_	TM_1_
I (3.881, 0.4017)	TE_0_	TE_1_	TE_2_	Hybrid	Hybrid
J (3.946, 0.4017)	TE_0_	TE_1_	TE_2_	TE_3_	TM_0_

At point A, fourth mode is TE_1_ which is indicated by γ_TE_ in maroon color. At point B in white-striped region, mode-4 is cutoff, as its effective index is below the BOX layer index of 1.443. In the blue region where point C is located, mode-4 is TM_1_ indicated by γ_TE_ close to 0. On the strip which is green–yellow, the mode is hybrid (between TE_2_ and TM_1_) which is indicated by point D. In the maroon strip where point E is located, mode-4 is TE_2_. Next region is again blue (sample point H), and mode-4 is TM_0_. The second green–yellow curve indicates hybrid between modes 4 and 5 (TE_3_ and TM_0_) and a sample point I is shown on this curve. Finally, γ_TE_ is maroon in the patch where point J is located, and mode-4 is TE_3_ here. Similar numerical analysis is carried out for X-cut Z-propagating LN waveguides and was observed that the hybrid region is narrow and steeper compared to the Y-propagating case.

**Figure 5:**
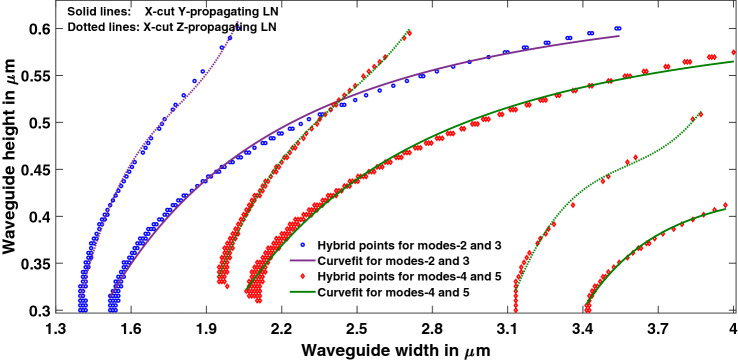
Curve fit for mode hybridization in X-cut Y-propagating and Z-propagating LNOI waveguides. [Matlab R2018a (9.4.0.813654), URL: www.mathworks.com].

The points (w, h) that lead to TE polarization fraction in the range of 0.4 to 0.6 are fitted with a Matlab curve fitting tool, shown in Fig. 5. Hybridization curves for Y-propagating LN are fitted with a two-term power series model that uses non-linear least-squares algorithm. The expressions obtained for representatives of the first two hybrid curves are given by Eq. (6), while the values of coefficients with 95% confidence bounds are specified in Eqs. (7) and (8). The goodness of the fits was also evaluated and was found that the root mean squared error is no more than 0.0073.

6 $$h = f\left( w \right) = aw^{b} + c$$

The coefficients for the first curve representing hybrid between TM_0_ and TE_1_ are,

7 $${\text{a }} = - \,0.{7915}\, \, \left( { - \,0.{8239}, - \,0.{7592}} \right);\quad {\text{b}} = - \,{2}.0{93}\, \, \left( { - {2}.{283}, - \,{1}.{9}0{2}} \right);\quad {\text{c}} = 0.{647}\, \, \left( {0.{6311}, \, 0.{663}} \right).$$

The coefficients for the second curve representing hybrid between TE_2_ and TM_1_ are,

8 $${\text{a}} = - \,{1}.{918}\,\left( { - \,{2}.{164}, - \,{1}.{673}} \right);\quad {\text{b}} = - \,{2}.{626}\,\left( { - \,{2}.{86}, - \,{2}.{392}} \right);\quad {\text{c}} = 0.{6138}\,\left( {0.{5997}, \, 0.{628}} \right).$$

Hybridization curves for Z-propagating LN are fitted with a linear polynomial of third order as shown in Fig. 5, expressions obtained for representatives of the first two hybrid curves are given by Eq. (9) and the coefficients are specified in Eq. (10),

9 $$h = f\left( w \right) = aw^{3} + bw^{2} + cw + d$$

Coefficients (with 95% confidence bounds):

10 $${\text{a}} = {1}.{7}0{4 }\,\left( {{1}.{115},{ 2}.{292}} \right);\quad {\text{b}} = - \,{9}.{115}\,\left( { - \,{12}.0{8}, - \,{6}.{148}} \right);\quad {\text{c}} = {16}.{53}\,\left( {{11}.{58},{ 21}.{48}} \right);\quad {\text{d}} = - \,{9}.{636}\,\left( { - \,{12}.{37}, - \,{6}.{9}0{5}} \right).$$

The fits are chosen to have least number of coefficients in standard fits with minimum root mean square error (RMSE). For example, the mode hybridization curves for Z-cut LN waveguides were fitted with second, third and fourth order polynomials which gave an RMSE of 0.01294, 0.0106 and 0.009987 respectively. It is observed that the RMSE decreased by just a factor of 0.0006 by using fourth order polynomial instead of third order polynomial. It was also verified that all the coefficients of the chosen fit contribute significantly and further reduction in the number of coefficients would reduce the accuracy of calculations.

In the range of widths (1.3–4 µm) and height (0.3–0.6 µm) considered, there are 119 hybrid points (between TE_1_ and TM_0_) for Y-propagating, and just 69 points for Z-propagating LN waveguides with vertical sidewalls. The maximum coupling factors ‘k’ obtained from Eq. (1) for hybrids between TM_0_ and TE_1_ are 4.6843 × 10^4^/m and 4.7768 × 10^4^/m in Y-propagating and Z-propagating LN respectively. Modal hybridization becomes stronger with the introduction of angled sidewalls^6^, and the coupling factor increased to 6.7623 × 10^4^/m and 7.6152 × 10^4^/m respectively for Y-propagating and Z-propagating LN. Also, the number of hybrid points rose to 264 and 259 in Y- and Z-propagating LN respectively.

### Effect of wavelength variation

**Figure 6:**
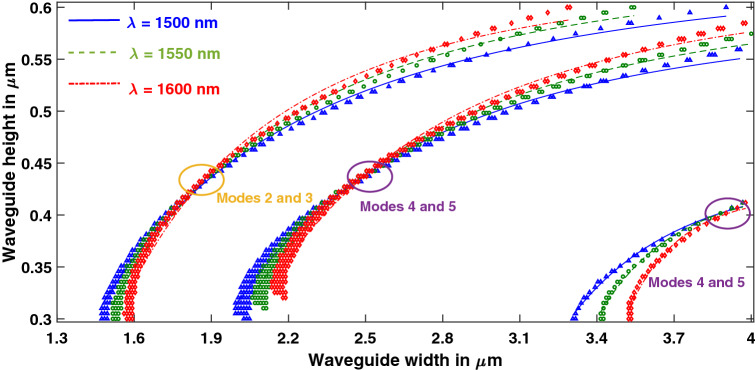
Effect of wavelength variation on hybrid points in Y-propagating LN. [Matlab R2018a (9.4.0.813654), URL: www.mathworks.com].

**Figure 7:**
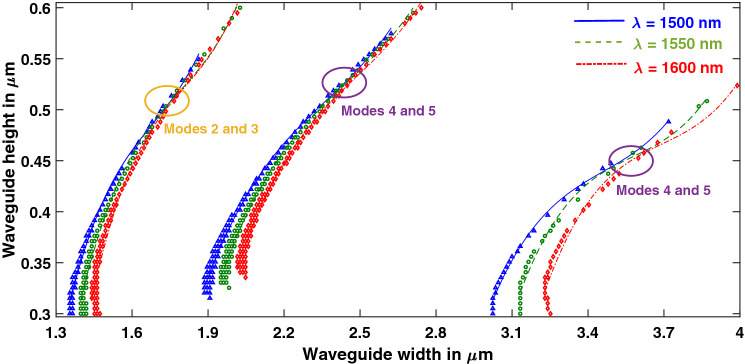
Effect of wavelength variation on hybrid points in Z-propagating LN. [Matlab R2018a (9.4.0.813654), URL: www.mathworks.com].

**Figure 8:**
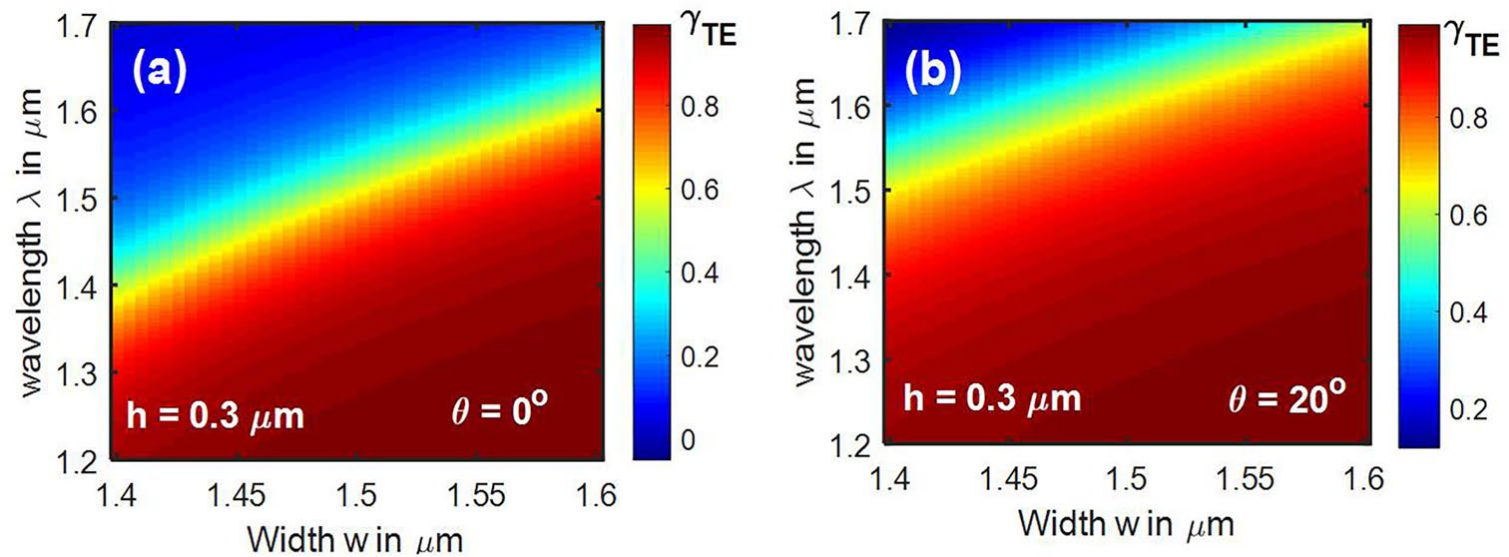
Effect of wavelength variation on hybrid points with sidewall angles of (**a**) θ = 0° (**b**) θ = 20°. [Lumerical 2020a MODE Finite Difference IDE, version: 7.15.2152, release: 2020a r2, URL: www.lumerical.com] [Image converted to JPG file with Adobe Photoshop, Version: 21.2.1 20200716.r.265, URL: www.adobe.com].

Hybrid points were extracted at wavelengths of 1500 nm, 1550 nm, and 1600 nm as shown in Figs. 6 and 7 for Y-propagating and Z-propagating LN respectively. Slope of the fitted curve tends to increase with the decrease in wavelength. Simulations were also performed with a fixed height of 0.3 µm for Y-propagating LN, wavelength spanning the telecommunication bandwidth, and width around the occurrence of hybrid mode between TM_0_ and TE_1_. At this particular height, hybrid region extended over a broadband of almost 50 nm as shown in Fig. 8a. However, there was no specific trend observed as the height was varied and other hybrid modes were evaluated.

### Effect of angled sidewalls

**Figure 9:**
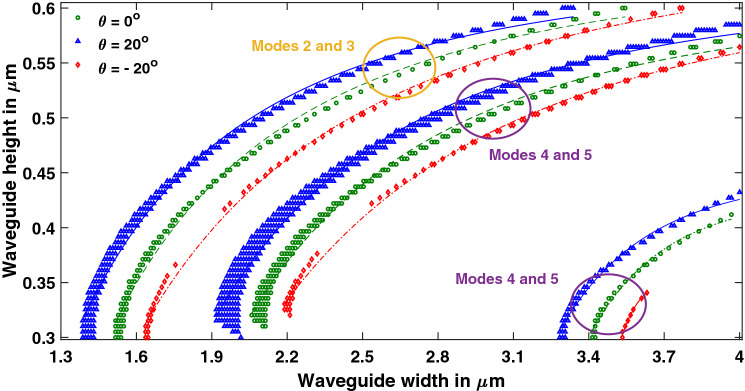
Effect of angled sidewalls on hybrid points of Y-propagating LN waveguide. At a fixed height, hybrid points shift towards lower widths with the positive sidewall angle. [Matlab R2018a (9.4.0.813654), URL: www.mathworks.com].

**Figure 10:**
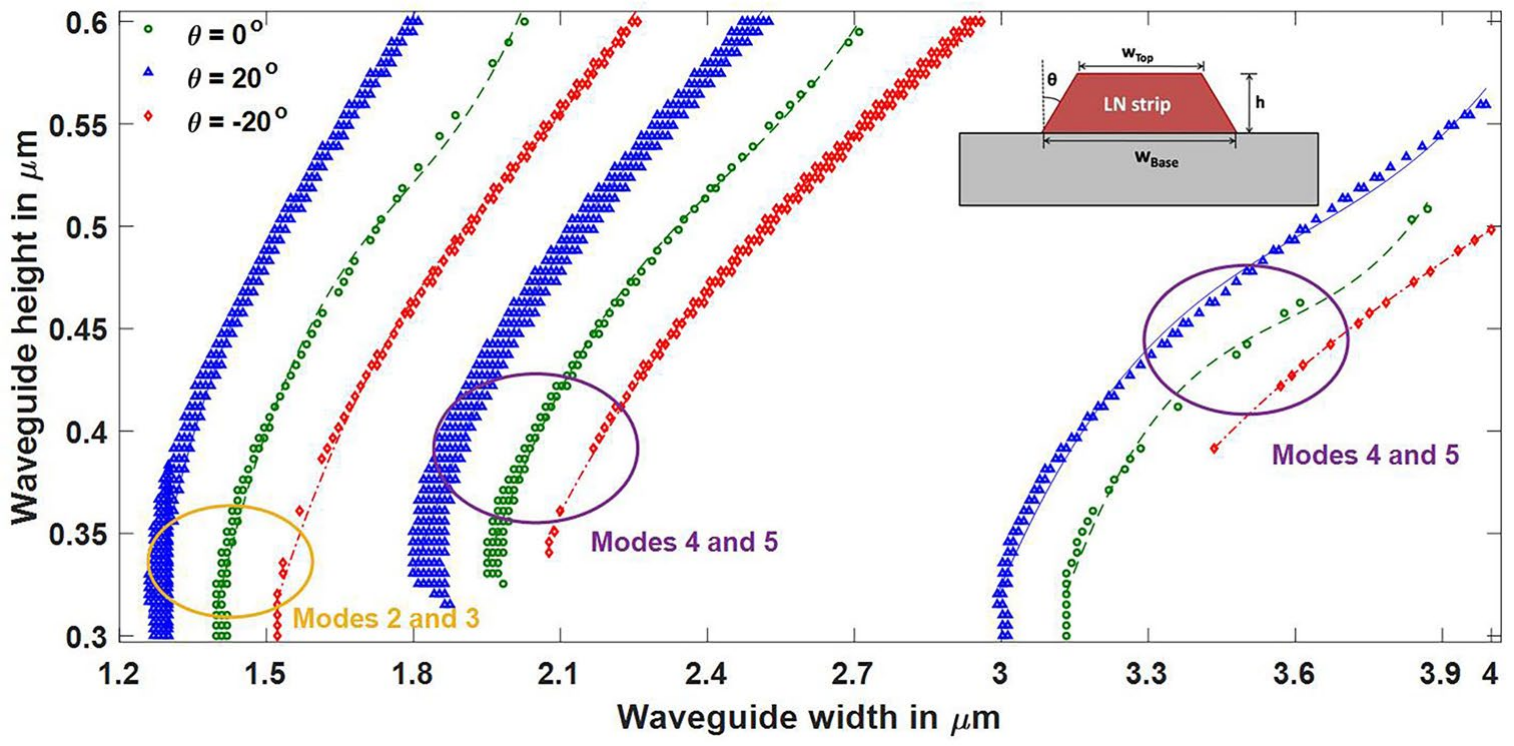
Effect of angled sidewalls on hybrid points of Z-propagating LN waveguide. At a fixed height, hybrid points shift towards lower widths with the positive sidewall angle. [Matlab R2018a (9.4.0.813654), URL: www.mathworks.com] [Image converted to JPG file with Adobe Photoshop, Version: 21.2.1 20200716.r.265, URL: www.adobe.com].

Lithium niobate is a difficult material to etch and angled sidewalls are a common feature of fabricated waveguides^25^. As a result, the points of hybridization (w_H_, h_H_) tend to shift towards different dimensions compared to waveguides with vertical sidewalls. Such shift in hybrid point of a particular mode was evaluated in^26^. In this work, the shift has been found for all hybrid points in the specified range of dimensions, at a side wall angle ‘θ’ of 20^o^ and − 20° as shown in Figs. 9 and 10.

As the sidewall angle increased at a fixed height, hybrid points shifted towards lower widths; at a fixed width, hybrid points shifted towards larger heights. Here, the top width is considered to be fixed, and the trapezoidal cross section of waveguide is formed by increase or decrease of bottom width. Increased bottom width leads to a positive sidewall angle and reduced bottom width leads to a negative sidewall angle. Angled sidewalls also increase the asymmetry of the waveguide and hence lead to stronger hybridness. This can be inferred from the increased density of points in left curve at θ = 20°, in each pair of the curves of Figs. 9 and 10. However, negative sidewall angle tends to reduce the hybrid points in both Y- and Z-propagating waveguides for heights lower than 0.4 µm. The effect of variation in wavelength on hybrid points in a waveguide with a sidewall angle of 20^o^ is shown in Fig. 8b, which indicates that band of hybrid points (cyan to yellow) shifted towards higher wavelengths. Recently, mode hybridization between fundamental TE and fundamental TM mode has been investigated in X-cut LNOI ridge waveguides, using its material birefringence^27^. Present work reports hybridization between TM_i_ and TE_i+2 k+1_ (i and k are integers), which can be extended to hybrids between TM_i_ and TE_i+2 k_ by introducing bends^27^ or using perturbation in the horizontal direction^23^.

### Mode hybridization in SOI strip waveguides

**Figure 11:**
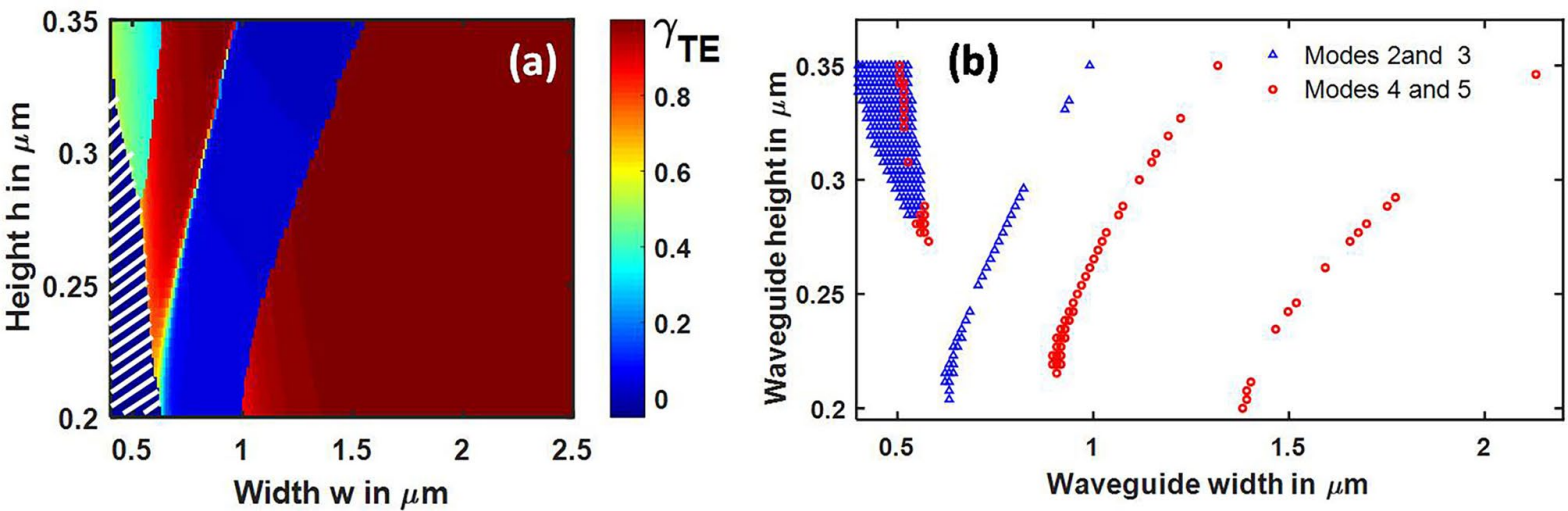
(**a**) TE polarization plot for hybrid between TM_0_ and TE_1_ in SOI strip waveguides (**b**) Mode hybridization regions in SOI strip waveguides. [11(a)-Lumerical 2020a MODE Finite Difference IDE, version: 7.15.2152, release: 2020a r2, URL: www.lumerical.com] [11(b)-Matlab R2018a (9.4.0.813654), URL: www.mathworks.com] [Image converted to JPG file with Adobe Photoshop, Version: 21.2.1 20200716.r.265, URL: www.adobe.com].

In order to compare the hybrid regions with that of LNOI, another popular platform of SOI is analyzed in this section. Silicon is isotropic and lacks intrinsic birefringence as opposed to LN. Mode hybridization regions are much narrower in SOI waveguides compared to the counterparts on X-cut LNOI. TE polarization plot for hybrid between TM_0_ and TE_1_ is shown in Fig. 11a and hybrid points between TM_0_–TE_1_ (modes 2 and 3), and TM_1_–TE_2_ (modes 4 and 5) are represented in Fig. 11b. The dense group of points forming a triangular shape on the top left corner majorly results from square-like core geometry. In this region, height of the waveguide approaches its width and results in modes with the horizontal and vertical electric field components becoming almost equal. Also, nodes increased to two in the vertical direction beyond a height of 270 nm.

### Design of tapers as mode converters based on mode hybridization

Mode converters are important for the implementation of mode division multiplexing schemes and polarization-independent devices. In this section, mode converters using tapers that utilize mode hybridization phenomena are designed on X-cut LNOI and analyzed numerically. The end widths of the taper are chosen such that w_1_ < w_H_ < w_2_, where w_H_ is the waveguide width for a specific mode hybridization region, and w_1_, w_2_ are taper end widths^6^.

### TM_0_ to TE_1_ mode converter

**Figure 12:**
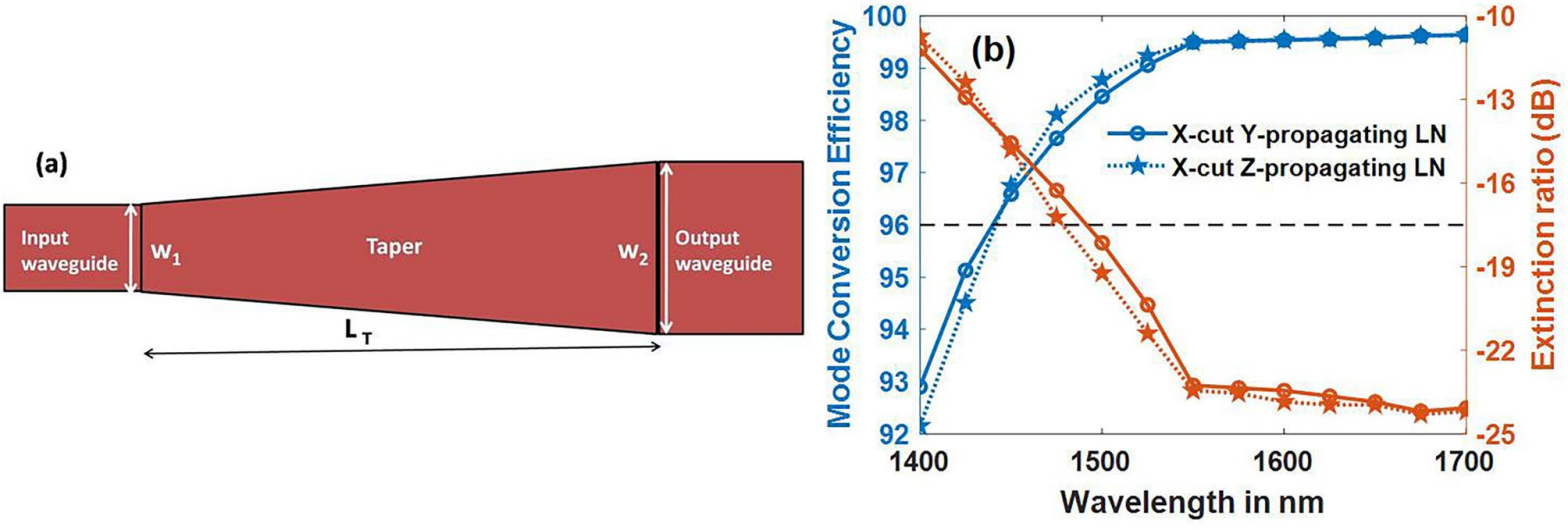
(**a**) Schematic of taper (**b**) dependence of MCE and ER on wavelength. [12(b)-Matlab R2018a (9.4.0.813654), URL: www.mathworks.com] [Image converted to JPG file with Adobe Photoshop, Version: 21.2.1 20200716.r.265, URL: www.adobe.com].

A taper is designed for conversion of TM_0_ to TE_1_ with a height of 0.4 µm, w_1_ < 1.725 µm < w_2_ (for Y-propagating) and w_1_ < 1.494 µm < w_2_ (for Z-propagating) at a wavelength of 1550 nm, whose schematic is shown in Fig. 12a. This choice of hybrid point (1.725 µm, 0.4 µm and 1.494 µm, 0.4 µm) was made from the data available in Fig. 5. Prominent figures of merit for this device are mode conversion efficiency (MCE), bandwidth (BW) and extinction ratio (ER). Mode conversion efficiency and extinction ratio^28^ are defined in Eqs. (11) and (12) and are plotted as a function of operating wavelength in Fig. 12b.

For an input TM mode,

11 $$MCE = \frac{{P_{TE} }}{{P_{TE} + P_{TM} }} \times 100\%$$

12 $$ER = 10log_{10} \left( {\frac{{P_{TM}^{out} }}{{P_{TE}^{out} }}} \right)$$

**Figure 13:**
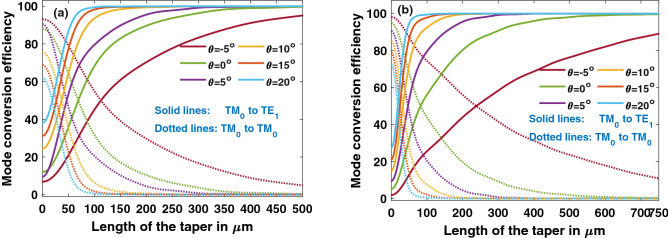
Mode conversion efficiency (MCE) of taper (TM_0_–TE_1_) with vertical and angled sidewalls in (**a**) Y-propagating LN (**b**) Z-propagating LN. MCE drops drastically for negative sidewall angles. [Matlab R2018a (9.4.0.813654), URL: www.mathworks.com].

To achieve a mode conversion efficiency of 99.5%, length of the taper was obtained as 477 µm and 707 µm in Y-propagating and Z-propagating waveguides respectively. MCE variation along the length of the taper is depicted in Fig. 13, and it is noticed that taper length (L_T_) that achieves required MCE comes down drastically with the increase in positive sidewall angle, due to stronger hybridization and coupling. From Figs. 9 and 10, it is known that hybrid points shift as the sidewall angle varies, and the change can be as large as 600 nm for a sidewall angle of 20° compared to vertical sidewall in both Y-propagating and Z-propagating LN. If the taper widths are chosen without considering shift in the hybrid points, there is an unnecessary increase in taper length to achieve the same efficiency, as tabulated in Table 2 (for 15° and 20°). Even small negative sidewall angle of 5° can almost double the taper length for desired efficiency.

**Table 2:** Taper length for a MCE of 99.5% with different θ at w_2_-w_1_ = 0.3 μm.

Sidewall angle θ (^o^)	L_T_ with shifts considered (μm)	L_T_ with shifts NOT considered (μm)	L_T_ with shifts considered (μm)	L_T_ with shifts NOT considered (μm)
	*Y-propagating LN*		*Z-propagating LN*	
0	477	–	707	–
5	328	304	329	342
10	216	181	197	185
15	135	208	142	185
20	102	212	104	188

**Figure 14:**
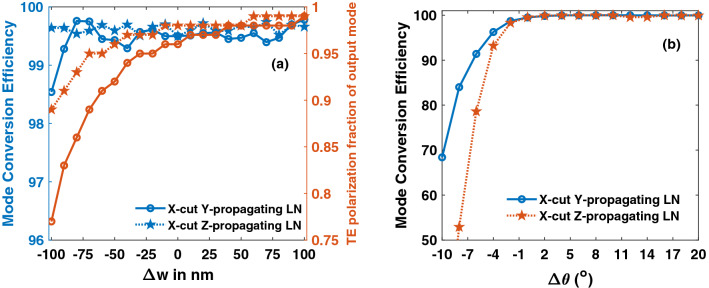
Fabrication tolerance to (**a**) waveguide width (**b**) sidewall angle. Mode converters on Y-propagating LN are more tolerant to negative sidewall angle variations. [Matlab R2018a (9.4.0.813654), URL: www.mathworks.com].

**Figure 15:**
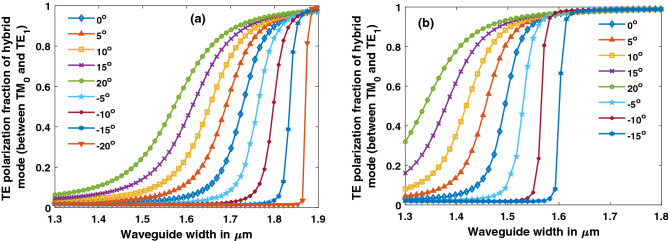
Dependence of TE polarization fraction of hybrid modes on width at different sidewall angles in (**a**) Y-propagating (**b**) Z-propagating LN. [Matlab R2018a (9.4.0.813654), URL: www.mathworks.com].

The device lengths are large compared to polarization converters on SOI counterparts, which has a length less than 100 µm^29^. This is obvious because of the large index contrast between core and clad in SOI waveguides. Very short device length of 15 µm is also reported^30^ on SOI platform that uses bilevel taper. As the fabrication technology of LN matures and losses come down, such designs can be attempted in LN as well. Cubic curving tapers^5^ could also be implemented to reduce the taper length. Moreover, LN comes with its own advantages of broad bandwidth of ~ 200 nm as compared to 100 nm in SOI^29^, and large fabrication tolerance as shown in Fig. 14. It is observed from Fig. 14a that MCE is stable for both Y-propagating and Z-propagating LN over a large width deviation (Δw) of − 100 to 100 nm. The difference between w_1_ and w_2_ is chosen to be 300 nm, and the hybrid width will remain well within this range even with Δw as large as ± 100 nm. It was observed that a smaller difference between w_1_ and w_2_ (δw) could lead to hybrid mode at the input end itself, as hybrid points are wide-spread in X-cut LNOI. Smaller δw can as well result in hybrid mode at the output if Δw is large due to fabrication errors. Figure 14a also shows the TE polarization fraction (γ_TE_), that is indicative of purity of output TE_1_ mode. At a Δw of − 100 nm, Y-propagating device has γ_TE_ of 0.77, while Z-propagating device has 0.89 for TE_1_ mode. Tolerance to sidewall angle is illustrated in Fig. 14b, and it is observed that both the configurations are stable for positive angles, but the effciency is highly sensitive to negative angles. To understand this better, dependence of TE polarization fraction of hybrid modes with the sidewall angle was evaluated at a fixed height as depicted in Fig. 15. Steep changes in TE polarization fraction at sidewall angles of − 5° and − 10° leaves a narrow choice of widths to choose from, for hybrid modes. This explains the reason for degradation of efficiency in Fig. 14b beyond an angle of − 4°.

**Figure 16:**
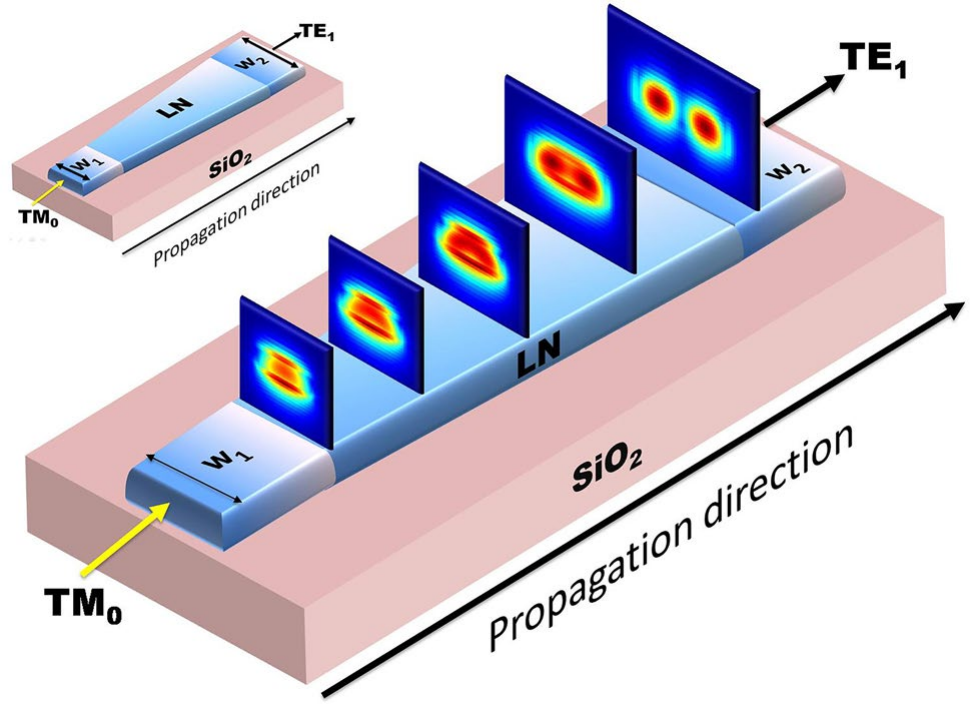
Mode profiles along the direction of propagation in Y-propagating LN taper for conversion from TM_0_ to TE_1_ mode. [Lumerical 2020a Finite Difference IDE, version: 8.23.2152, release: 2020a r2, URL: www.lumerical.com] [Image converted to JPG file with Adobe Photoshop, Version: 21.2.1 20200716.r.265, URL: www.adobe.com].

Simulations were performed using 3D FDTD method to accurately model the propagation of light through Y-propagating taper of length 477 µm. With TM_0_ mode launched through the input waveguide, TE_1_ was obtained at the output waveguide. Mode profiles at the intermediate points along the propagation direction is shown in Fig. 16. Long tapers which do not support mode conversion can also be designed using the results from Fig. 5, 9 and 10. The end widths w_1_ and w_2_ are to be chosen such that no hybrid point exsists in the range of widths between them.

## Conclusion

Mode hybridization in air-cladded strip multimode waveguides on X-cut LNOI and SOI is analyzed numerically to obtain mathematical relation between structural parameters leading to hybrid modes. Relation between width and height that lead to TE polarization fraction of 0.4–0.6 (which is a measure of hybridization) is found to be a two-term Power series in Y-propagating and 3^rd^ order polynomial in Z-propagating LN strip waveguides. Regions of hybridization were found to be wider in Y-propagating LN compared to Z-propagating LNOI and SOI counterparts. Hybridization regions occur at lower widths in the isotropic Z-propagating configuration comparaed to the birefringent Y-propagating configuration. Hybrid points tend to shift as the sidewall angle of the waveguide increased, and it could be as large as ~ 600 nm for a sidewall angle of 20° in comparison with vertical sidewalls. Waveguides with larger positive sidewall angles exhibited denser regions of hybridization as a result of enhanced asymmetry and higher coupling factors. Hybrid mode regions were utilized to design tapers that convert TM_0_ to TE_1._ To achieve MCE of 99.5%, length of the taper required is 477 µm in Y-propagating LN and 707 µm in Z-propagating LN, when the sidewalls are vertical. Tapers designed with the incorporation of shift due to angled sidewalls (> 10^o^) were found to achieve the specified MCE at a shorter length (reduced by 100 μm in Y-propagating and 84 μm in Z-propagating when θ = 20°). Even small negative sidewall angle of 5° can almost double the taper length for desired efficiency. Tapers on LN are found to exhibit a broad BW of ~ 260 nm with MCE greater than 96%, ~ 200 nm with extinction ratio less than − 18 dB; large fabrication tolerance of ± 100 nm and good extinction ratio of − 23 dB at design wavelength. These results of hybrid mode analysis are crucial for the design of tapers as mode converters, polarization rotators, multimode photonics, tunable time delays and optical signal processing applications. The results can as well be used to avoid hybrid modes, while designing multimode waveguides as optical interconnects and MDM demultiplexers in order to avoid unwanted mode coupling and crosstalk.

## Methods

TE polarization fraction values and effective indices of thin film lithium niobate waveguides are obtained using full-vectorial finite-difference Eigen mode solver from a commercial simulation tool (Lumerical). Sellmeier equation is used to model the dispersive nature of LN with diagonal anisotropy being incorporated in the material. LN is a negative uniaxial material, with the ordinary index n_o_ = 2.21116 and extraordinary index n_e_ = 2.13755 at a wavelength of 1550 nm. Eigen mode expansion method (EME) is used to analyze mode conversion in the taper. EME is a frequency-domain method for solving Maxwell’s equations that is ideal for simulating light propagation over long distances. This is chosen over FDTD for the benefits of speed and low computational cost. Simulations were also performed using 3D FDTD method to accurately model the propagation of light through taper that achieves desired mode conversion.
